# Mental health of Korean adults before and during the COVID-19 pandemic: a special report of the 2020 Korea National Health and Nutrition Examination Survey

**DOI:** 10.4178/epih.e2022042

**Published:** 2022-04-25

**Authors:** Hyunsuk Jeong, Suyeon Park, Jihee Kim, Kyungwon Oh, Hyeon Woo Yim

**Affiliations:** 1 Department of Preventive Medicine, College of Medicine, Catholic University of Korea, Seoul, Korea; 2 Division of Health and Nutrition Survey and Analysis, Bureau of Chronic Disease Prevention and Control, Korea Disease Control and Prevention Agency, Cheongju, Korea

**Keywords:** COVID-19, Stress, Depression, Suicide, Mental health, Korea National Health and Nutrition Examination Survey

## Abstract

**OBJECTIVES:**

Coronavirus disease 2019 (COVID-19) and the associated social distancing, limited freedom, and fear of an uncertain future are expected to have substantial mental health effects. We investigated mental health responses in the community during the first year of the COVID-19 pandemic in Korea.

**METHODS:**

We used 2016-2019 and 2020 data from the Korea National Health and Nutrition Examination Survey (KNHANES) to assess pre-pandemic and pandemic mental health status, respectively, in terms of perceived severe stress, depression, and suicidal plans. All analyses were gender-stratified. Pre-specified subgroup analyses were performed according to age, employment status, and household income.

**RESULTS:**

The percentage of Korean adults with suicidal plans increased significantly from 1.3%p (95% confidence interval [CI], 1.1 to 1.5) in 2016-2019 to 1.8%p (95% CI, 1.4 to 2.1) in 2020. Individuals in their 20s and 40s showed a marked increase in suicidal plans (1.2%p; 95% CI, 0.0 to 2.3 and 0.9%p; 95% CI, 0.0 to 1.8, respectively). In men, depression and perceived severe stress increased significantly from pre-COVID-19 to 2020. There was a 2.4%p (95% CI, 0.8 to 4.0) increase in depression among standard workers and a 2.9%p increase in depression in individuals in the second-highest quintile of household income from 2016 and 2018 to 2020.

**CONCLUSIONS:**

As COVID-19 continued, mental health issues such as suicidal plans, depression, and severe stress increased significantly in young men and people in the second-highest quintile of household income. Proactive community mental health efforts are needed to prevent increases in the suicide rate resulting from prolonged exposure to the COVID-19 pandemic.

## GRAPHICAL ABSTRACT

[Fig f1-epih-44-e2022042]

## INTRODUCTION

Coronavirus disease 2019 (COVID-19) first emerged in Wuhan, China in December 2019. The virus rapidly spread around the world, prompting the World Health Organization to declare it a global pandemic on March 11, 2020 [1]. In Korea, a COVID-19 outbreak was declared on January 20, 2020. In response to this global health crisis, the Korean government implemented several measures to control COVID-19, including social distancing, mandatory use of face masks indoors, avoidance of large gatherings, travel restrictions, teleworking, online classes for students, and orders to stay at home.

The COVID-19 pandemic has disrupted people’s daily life. Movement restrictions, separation from family or friends, limited freedom, and fear of an uncertain future are all factors that may have a negative psychological impact [2]. Social distancing and restrictions due to the COVID-19 are expected to have substantial negative mental health effects, including increased depression, anxiety, acute stress, and insomnia [3].

A previous study reported that COVID-19 was associated with mental illness among healthcare workers in China exposed to patients with COVID-19 [4]. Medical students and medical staff treating patients with COVID-19 reported an elevated prevalence of anxiety and stress [5,6]. However, most previous findings have focused on specific subpopulations, such as medical students and healthcare workers.

Studies comparing mental health outcomes before the pandemic with results obtained during the early weeks of the pandemic have shown an increase in clinically significant levels of psychological distress [7]. During the COVID-19 pandemic, many individuals have experienced a wide range of adversities, including challenges with meeting basic needs, increased caregiving responsibilities, difficulties with accessing non-COVID-19-related healthcare, employment and financial loss, and disruption of social networks, all of which can increase the risk of mental illness [8].

Several cross-sectional studies have evaluated the mental health impact of COVID-19 in Korea during the acute period of the COVID-19 pandemic. Clinical levels of depression, anxiety, or stress were reported by 45% of respondents [9]. According to another report, 18.8% of the participants had depressive symptoms, 10.6% had anxiety symptoms, and 5.1% had a high level of perceived stress during the COVID-19 pandemic [10]. A similar increase in distress was observed in a longitudinal community-based prospective cohort study known as the Cardiovascular and Metabolic Etiology Research Center study [11]. Those results also showed that there were no significant gender differences in depression, anxiety, post-traumatic stress disorder, and loneliness at 55 days after the start of the COVID-19 outbreak [11].

The ongoing pandemic and associated social isolation could have a huge impact on individuals’ mental health. Consequently, it is crucial to understand the extent of the impact of the pandemic on the mental health of the general population. According to the COVID-19 National Mental Health Survey conducted by the Korean Society of Traumatic Stress Studies, the prevalence of depression and anxiety during the first year of the COVID-19 pandemic was 20% and 15%, respectively [12]. A similar increase in distress was observed in a longitudinal study of adults in the United Kingdom [13]. Studies in China [14] and Switzerland [15] have also found an increase in depression during the early stages of the pandemic.

However, the previous findings in the Korean population were derived from web-based surveys and had an online modality with a risk of selection bias. There may be limitations in generalizing the results of limited samples to the entire Korean population [9-11]. Since the COVID-19 National Mental Health Survey has been investigating depression, anxiety, stress, and suicide risk on a quarterly basis since 2020, it might allow observations of changes in mental health status in accordance with the spread of COVID-19 [12]. However, a limitation is that its findings cannot be compared to before the COVID-19 pandemic. In contrast, since the Korean National Health and Nutrition Examination Survey (KNHANES) has been conducting surveys annually with the same sampling frame, survey tools, and methods, data from the KNHANES can be used to identify changes in mental health status in the community after the COVID-19 pandemic with reference to the pre-pandemic status.

In the current study, we investigated the magnitude of increases in perceived severe stress, depression, and suicidal plans as mental health outcomes pre-pandemic (2016-2019) and during the first year of the COVID-19 pandemic (2020) using the KNHANES data in order to determine the impact of the first year of COVID-19 on the mental health of the general population of Korea.

## MATERIALS AND METHODS

### Study population

The KNHANES, a population-based survey designed to collect information on the health and nutrition of the Korean household population, has surveyed a nationally representative subset of the civilian population of Korea since 1998 using a complex, stratified, multistage probability sampling design [16]. In this context, the primary sample units (PSUs) for KNHANES are selected from a sampling frame of all census blocks or resident registration addresses. Each PSU consists of approximately 50 households to 60 households. Following the selection of PSUs, all dwelling units in the PSU are listed and 23-25 households are selected for a field survey for household screening. The final stage of selection occurs in the household, where all members aged 1 year or older are selected to participate. To assess pre-pandemic mental health status, we used a KNHANES (2016-2019) sample of 24,502 individuals to evaluate perceived severe stress and suicidal plans and a KNHANES (2016, 2018) sample of 11,679 individuals to evaluate depressive symptoms. A KHANES 2020 sample of 5,857 individuals was included to evaluate the effects of the first year of the pandemic on mental health. The analysis was limited to participants who were 19 years of age or older. Differences in severe perceived stress, depression, and suicidal plans before and during the COVID-19 pandemic were analyzed separately. A flowchart of the samples included in the analysis for each outcome is presented in Supplementary Material 1.

### Measurements

Mental health problems were assessed by self-reported stress perception, depressive symptoms, and suicidal plans. The level of perceived stress was measured using the following question: “How much stress do you usually feel?” Four response options were provided: “low,” “middle-low,” “middle-high,” and “extreme” stress. According to the guideline of KNHANES data analysis (2016-2018), stress perception was classified into 2 categories: high (either extreme stress or middle-high) and low (middle-low or low). In the current study, however, stress perception was classified into 2 categories: extreme (extreme stress) and not extreme (middle-high, middle-low, or low). Since the pandemic has lasted for over a year, a severe stress level is considered an appropriate indicator of mental health. People who reported high levels of stress were defined as being under severe perceived stress. Perceived stress was assessed annually.

Depression was evaluated using Patient Health Questionnaire-9 (PHQ-9). The PHQ-9 is a self-reporting assessment tool that is useful for depression screening and can be used to determine the severity of depressive symptoms [17]. The KNHANES administered the PHQ-9 for the first time in 2014, and it has been administered every other year ever since then. It consists of a 9-item depression module that assesses whether individuals experience little interest, hopelessness, sleep problems, changes in appetite, fatigue, a sense of guilt or worthlessness, concentration difficulties, moving or speaking very slowly or the opposite, and/or suicidal ideation over the past 2 weeks; these items were taken directly from the criteria of Diagnostic and Statistical Manual of Mental Disorders, 4th edition (DSM-IV). Each item is scored from 0 (not at all) to 3 (almost every day); thus, a higher combined score indicates a higher severity of depression. Clinically significant depression requiring treatment was determined using an optimal cut-off score of 10 [17,18]. The PHQ-9 has previously been validated for use in the Korean population [19].

An individual was considered to have suicidal plans if he or she answered “yes” to the following question: “Have you made a plan to commit suicide within the last year?” Suicidal plans were assessed annually in the same manner. The presence of suicide plans during the past year was derived from the Structured Clinical Interview for DSM-IV Axis I Disorders (SCID-I) [20]. The Korean version of the SCID showed moderate to excellent inter-rater agreement and was recommended as an accurate diagnostic tool in clinical practice and research on psychiatric disorders [21].

### Demographic characteristics

Participants reported their age (grouped into 19-29, 30-39, 40-49, 50-59, 60-69, and ≥70 year-olds), gender (men, women), monthly household income (stratified into 5 groups, with the first quintile corresponding to the lowest income quintile and the fifth quintile being the highest income quintile).

Employment was classified into 4 categories by asking the following 2 questions: “Which of the following best describes your work?” Possible answers included wage worker, self-employed, unpaid family worker, housewife, and student. Those who responded that they were wage workers were additionally asked the following question: “Are you currently employed as a standard worker (permanent or tenured employee) or non-standard worker (temporary or part-time worker)?” Those who responded “self-employed” to the initial question were categorized as employers. Standard workers were defined as those who responded that they were wage workers in the initial question and standard workers in the additional question. Non-standard workers were defined as those who responded that they were wage workers in the initial question and non-standard workers in the additional question. The unemployed were defined as those who responded that they were unpaid family workers, housewives, or students in the initial question.

### Statistical analysis

The COVID-19 pandemic has been reported to affect people disproportionately depending on their gender; therefore, we stratified mental health effects by gender. Pre-specified subgroup analyses were performed according to age, employment status, and household income. The categorical variables are expressed as numbers and weighted percentages. All comparisons and analyses were weighted to account for the complex survey design of both studies and to produce nationally representative estimates. Sampling weights were generated by considering the complex sample design and non-response rate of the target population. The weighted prevalence and confidence intervals were calculated using PROC SURVEYFREQ, and the weighted difference and confidence intervals between the 2 periods were calculated using PROC SURVEYREG. All statistical analyses were analyzed by 2-tailed tests, and the results were considered statistically significant for p-value < 0.05 using SAS version 9.4 (SAS Institute Inc., Cary, NC, USA).

### Ethics statement

The KNHANES is a research endeavor conducted by the government for public welfare in accordance with paragraph (1) of Article 2 of the Bioethics and Safety Act and Article 2 (2) 1 of the Enforcement Rule of the same act; it is therefore deemed exempt from review by the institutional review board (IRB), and the KNHANES 2016-2017 was conducted without IRB approval. Since the KNHANES 2018, IRB approval after review has been obtained since the KNHANES collects human-derived material, and the KNHANES raw data have been available to third-party users for KNHANES 2018-2020 (2018-01-03-P-A, 2018-01-03-C-A, and 2018-01-03-2C-A). All subjects signed a consent form before participating in the survey.

## RESULTS

The socio-demographic characteristics of the KNHANES participants in 2016-2019 and in 2020 are shown in Table 1. The total number of subjects aged 19 years or older was 24,856 in 2016-2019 and 5,922 in 2020. Of those, 10,927 were men and 13,929 were women in 2016-2019; 2,651 were men and 3,271 were women in 2020. The gender ratio was the same for each survey period. For both men and women, the distributions of age group, employment status, and household income were similar between the 2016-2019 and 2020 surveys (Table 1).

The weighted prevalence of perceived severe stress for the total population increased from 4.9% (95% confidence interval [CI], 4.6 to 5.2) in 2016-2019 to 5.4% (95% CI, 4.6 to 6.2) in 2020, although this difference was not significant. In subgroup analyses, significant differences were found in perceived severe stress between before and during the pandemic among all men, men in their 30s-40s, and standard workers. In men, the weighted prevalence of perceived severe stress increased from 4.1% (95% CI, 3.7 to 4.5) in 2016-2019 to 5.4% (95% CI, 4.2 to 6.6) in 2020. The 1.3%p (95% CI, 0.0 to 2.5) increase in perceived severe stress from 2016-2019 to 2020 was statistically significant. Perceived severe stress rose most sharply among adults aged 30-39 years and 40-49 years, increasing from 5.5% and 4.0% in 2016-2019 to 9.5% and 7.2% in 2020, respectively, corresponding to statistically significant differences of 4.0%p (95% CI, 0.0 to 8.0) and 3.2%p (95% CI, 0.3 to 6.1), respectively. The 2.2%p (95% CI, 0.1 to 4.4) increase in perceived severe stress from 2016-2019 to 2020 was statistically significant among standard workers. However, perceived severe stress did not differ pre-pandemic and during the first year of the pandemic among women (Table 2).

The weighted prevalence of depression as detected by PHQ-9 increased from 4.9% (95% CI, 4.4 to 5.4) in 2016 and 2018 to 5.3% (95% CI, 4.5 to 6.1) in 2020, but this difference was not significant for the total population. In subgroup analyses, there were significant differences in PHQ-9 depression pre-and post-pandemic among all men, men in their 30s, standard workers, and those with household income in the fourth quintile. In men, the prevalence of depression increased from 3.2% (95% CI, 2.6 to 3.8) in 2016 and 2018 to 4.4% (95% CI, 3.4 to 5.4) in 2020. The 1.2%p (95% CI, 0.0 to 2.3) increase in depression from 2016 and 2018 to 2020 was statistically significant. Depression rose most sharply among adults aged 30-39 years, increasing from 3.6% in 2016 and 2018 to 6.5% in 2020, a statistically significant difference of 3.0%p (95% CI, 0.0 to 6.0). The 2.4%p (95% CI, 0.8 to 4.0) increase in depression from 2016 and 2018 to 2020 was statistically significant among standard workers. Moreover, depression significantly increased by 2.9%p (95% CI, 0.6 to 5.1) in those who were in the fourth quintile of household income. In women, there was no significant difference in depression before and after the pandemic regardless of age, household income, or employment status (Table 3).

The weighted prevalence of suicidal plans increased from 1.3%p (95% CI, 1.1 to 1.5) in 2016-2019 to 1.8%p (95% CI, 1.4 to 2.1) in 2020. The 0.5%p (95% CI, 0.1 to 0.9) increase in suicidal planning from 2016-2019 to 2020 was statistically significant. In the subgroup analyses, individuals in their 20s and 40s, and those in the fourth quintile of household income showed significant differences in the prevalence of suicidal plans between before and during the pandemic. The prevalence of suicide plans rose most sharply among adults aged 19-29 years, increasing from 1.2% in 2016 and 2018 to 2.4% in 2020, a statistically significant difference of 1.2%p (95% CI, 0.0 to 2.3). In individuals aged 40-49 years of age, the 0.9%p (95% CI, 0.0 to 1.8) increase in suicidal planning from 2016-2019 to 2020 was statistically significant. The prevalence of suicidal plans also increased significantly by 0.8%p (95% CI, 0.0 to 1.5) in those who were in the top fourth quintile of household income. However, there was no significant difference in suicidal plans when stratified by gender (Table 4).

## DISCUSSION

As COVID-19 continued, mental health issues such as suicidal plans, depression, and severe stress increased significantly in young men and people in the second-highest quintile of household income.

Our findings indicate that the weighted prevalence of suicidal plans increased by over 40% from before the pandemic to the first year of the pandemic; in particular, it doubled among those in their 20s and 40s. The increase in the prevalence of suicidal planning observed among young adults is comparable to that observed in studies of young adults in the United Kingdom and United States [22,23]. The financial insecurity and job loss experienced by many young adults due to COVID-19 may have contributed to the sustained rise in suicidal behaviors observed in this study [24]. Individuals in their 40s are the heads of households, and it is possible that the increased prevalence of suicide planning in this age bracket may reflect the burden of having responsibility for the family in economically difficult times. This psychological burden may manifest as suicide plans. In Japan, the percentage of suicidal behavior by age group between January and November 2020 was the highest in individuals in their 40s, at 17.4% [25].

There are gender differences in reactions to COVID-19. More adverse mental health issues, including increased depression and perceived severe stress, were observed in men than in women. The weighted prevalence of depression increased by 40%, and perceived severe stress increased by 30% from before the pandemic to the first year of the pandemic among men; although women had a higher prevalence of depression than men, there was no difference before and after the pandemic.

In the 1970s, Weissman first highlighted gender differences in depression and pointed out that in clinical and community samples, about twice as many women suffered from depression as men [26]. Since then, the theory of gender differences in depression has surged. Epidemiological findings point to a women preponderance in the prevalence of depression [27-29]. Similarly, depression was higher in women than in men for most age groups in Korea [30]. The point prevalence of depression among women was higher than that of men both in cross-sectional studies that were conducted during the acute period of the COVID-19 pandemic and in regular mental health assessment surveys conducted during the COVID-19 pandemic. Nonetheless, although the point prevalence of depression was higher in women than in men, the prevalence of depression in men showed a statistically significant increase during the COVID-19 pandemic.

The number of hours spent on childcare has increased due to an increase in flexible work arrangements and school closure during the pandemic in United Kingdom men [31]. Similarly, housework and especially childcare seem to have become equally shared between men and women in Canada [32]. In line with this evidence, a more gender-equal share of additional childcare activities was observed following the Italian lockdown [33]. In the same way in Korea, as the time spent at home increases during the pandemic, the burden of housework and childcare for men will be greater than before the pandemic. The increase in housework burden may have acted as a factor that increased men’s depression and stress.

By age group, depression in men in their 30s nearly doubled and perceived severe stress in men in their 30-40s increased by almost 80% during the first year of the pandemic compared to the pre-pandemic period. Aging increases the risk of COVID-19 infection and mortality; however, studies have shown that during the pandemic, levels of depression and stress were significantly higher in the age group of 21-40 years than in older age groups [34,35]. The main reason for this seems to be that individuals in this age range are concerned about the future and economic challenges caused by the pandemic due to their roles as key active working members of society, who are therefore most strongly affected by redundancies and business closures.

Among men standard workers, weighted severe stress levels increased by 60% and depression doubled from pre-pandemic to the first year of the pandemic. In the cross-sectional results for men’s depression prevalence, a higher proportion of depression was found in non-standard workers than in standard workers both pre-pandemic and during the pandemic. Compared with pre-COVID-19, however, the prevalence of depression among standard workers during the first year of COVID-19 demonstrated a statistically significant increase. Before the COVID-19 pandemic, men standard workers had the lowest prevalence of depression, but the sharp increase in the first year of COVID-19 seems likely to be interpreted as reflecting the impact of the COVID-19 situation.

As COVID-19 reemerged in metropolitan areas, stricter social distancing measures (level 2), including shortened business hours, were implemented in Korea, although these policies were not as restrictive as lockdowns in the European Union or the United Kingdom. Nonetheless, these imposed restrictions, as well as people voluntarily refraining from everyday activities, led to many businesses experiencing reduced demand for goods, resulting in an economic recession. Moreover, an unpaid leave policy was instituted for employees in various professional settings such as airlines and travel agencies [36]. These policies would have added to the psychological burden of standard workers.

The COVID-19 pandemic also resulted in an employment crisis with disruption of production and the labor market. The explosion of unemployment became so acute in March and April 2021 that the Korean government imposed level 2 quarantine measures, including social distancing and restriction of social gatherings. More than 1 million individuals lost their jobs in a month [37]. Ironically, as the ‘untact’ economy (e.g., e-commerce companies) has rapidly expanded, the number of platform workers associated with delivery services has also drastically increased [36]. This industrial structure has increased the number of nonregular workers, and regular workers face uncertainty as to when their employment status will normalize. This may explain why perceived severe stress and depression among men standard workers increased during the first year of the pandemic.

We chose 3 mental health indicators (perceived stress, depression, and suicidal plans) as outcomes. We supposed that suicide attempts and suicide deaths would not show impacts during the first year of COVID-19, as there was a mix of hope and uncertainty that COVID-19 would soon end. According to a previous report on disasters and mental health, suicide rates in communities due to disasters were reported to increase years later, not immediately after a disaster [38]. Victims would take on loans to recover from the disaster. It is possible that loans delay some of the psychological consequences by bringing temporary relief to the victims, but leave financial burdens long after everything seems to have returned to normal. This phenomenon is expected to be similar to the crisis that Korean society will face due to the prolonged COVID-19 pandemic. In fact, the suicide rate in Korea did not increase during the first year of COVID-19. Generally, suicidal ideation presents in a “waxing and waning manner,” so the magnitude and characteristics of suicide ideation fluctuate dramatically, and suicidal ideation is considered a better predictor of lifetime risk for suicide than a predictor of imminent risk [39]. Since extreme stress, depression, and suicidal ideation are strong predictors of suicide attempts or death by suicide, we compared extreme stress, depression, and suicide planning before COVID-19 versus the first year of COVID-19.

This study has several limitations. We did not standardize comparisons between the pre-pandemic period (2016-2019) and the first year of the pandemic (2020). However, because 2016-2020 is a relatively short period, we did not expect changes in the population composition during this period, and all frequencies were calculated as weighted values for a representative Korean population in the current analyses. Moreover, it is important to note that our study sample did not include individuals residing in long-term care institutions, which may have led to underestimation of the mental health problems of the general population, limiting the generalizability of our findings to community-dwelling populations only. To prevent the spread of infectious diseases, family visits to inpatients in long-term care institutions were restricted. Individuals residing in long-term care institutions have experienced a reduction in social contacts, with a consequent increase in social isolation and feelings of loneliness, which are in turn associated with increased severe stress, depression, and suicidal ideation.

To understand these trends, it is preferable to consider temporal patterns or trends in perceived stress, depression, and suicide plans before COVID-19. However, we presented the pre-COVID-19 data as the average fraction of participants with mental health problems from 2016 to 2019 for 2 reasons. First, the proportion of mental health variables between 2016 and 2019 was similar. Second, as depressive symptoms were surveyed every other year, there were insufficient temporal data to see a trend.

Perceived stress was measured as a single item and is not a tool developed through standardization. Although the question has not been validated, we used standardized measurements of depression and suicide plans as mental health indicators.

A strength of this study is that we drew on nationally representative probability based samples that included the same measures at each time point, enabling the generation of meaningful estimates of population prevalence. In addition, we tested whether the pandemic is likely to have disproportionately affected specific population subgroups.

It should be noted that during previous infectious disease outbreaks, suicide prevalence has been found to be elevated. Specifically, suicide rates increased during the Spanish Flu of 1918-1919 in the United States, during the severe acute respiratory syndrome outbreak in China, as well as during the Ebola infection in Africa [40-42]. According to cause of death data from the National Statistical Office, the number of suicides in Korea decreased by 4.4% in 2020 compared with 2019 [43]. The pandemic, despite many anticipated negative impacts on mental health, may not lead to an immediate increase in the suicide rate. However, it is possible that the long-term negative social and economic impacts of the COVID-19 pandemic may result in an increase in the suicide rate. As suicide planning has increased during the first year of the pandemic and suicide planning is a strong predictor of suicide attempts or suicide-related deaths [44], policy efforts are required to ensure that prolonged COVID-19 does not lead to an increased suicide rate.

Public health and social measures for COVID-19, which involve various restrictions in daily life and social distancing, are emotionally challenging [45]. Economic conditions are expected to decline, which will pose a heavy burden for many people. These conditions can negatively affect mental health, which can increase the risk of suicide [45,46].

## CONCLUSION

As the infectious disease crisis continues, the prevalence of suicidal plans, depression, and severe stress increased substantially, even in young men and the economically privileged, although these effects did not directly translate into an increased number of deaths by suicide. However, given that the mental health effects of the pandemic are likely to increase over time due to prolonged economic stress, underemployment, or post-infection sequelae, we anticipate an increase in the prevalence of some psychiatric illnesses. Proactive community mental health efforts are needed to address the detrimental mental health effects of the prolonged COVID-19 pandemic.

## Figures and Tables

**Figure f1-epih-44-e2022042:**
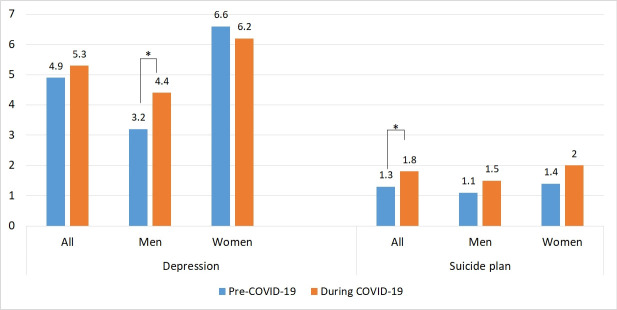


**Table 1.** Socio-demographic characteristics in the KNHANES 2016-2019 and 2020 samples stratified by gender

Characteristics		Men		Women	
		2016-2019 (n=10,927)	2020 (n=2,651)	2016-2019 (n=13,929)	2020 (n=3,271)
Age (yr)					
	19-29	1,402/9.4 (8.8, 10.0)	400/9.0 (7.9, 10.1)	1,544 /8.3 (7.8, 8.8)	401/8.3 (7.4, 9.2)
	30-39	1,717/9.2 (8.6, 9.8)	339/8.7 (7.4, 9.9)	2,132/8.5 (8.0, 9.0)	430/7.9 (6.9, 8.8)
	40-49	1,988/10.2 (9.7, 10.7)	423/9.7 (8.7, 10.8)	2,552/9.9 (9.4, 10.3)	550/9.3 (8.3, 10.3)
	50-59	2,004/10.0 (9.5, 10.4)	485/9.9 (8.9, 10.9)	2,705/9.9 (9.5, 10.3)	580/9.8 (9.0, 10.6)
	60-69	1,929/6.4 (6.1, 6.8)	488/7.2 (6.4, 8.1)	2,435/6.8 (6.4, 7.1)	636/7.6 (6.8, 8.4)
	≥70	1,887/4.6 (4.3, 4.9)	516/5.1 (4.4, 5.8)	2,561/6.9 (6.5, 7.3)	674/7.4 (6.5, 8.4)
Employment status					
	Employer	2,259/9.6 (9.1, 10.1)	513/9.4 (8.4, 10.4)	1,183/4.1 (3.9, 4.4)	293/4.6 (4.0, 5.3)
	Standard worker	2,976/15.7 (15.0, 16.4)	675/14.7 (13.4, 16.0)	1,865/7.4 (7.0, 7.9)	479/8.1 (7.2, 8.9)
	Non-standard worker	2,047/9.6 (9.1, 10.1)	488/9.6 (8.6, 0.5)	3,299/12.5 (12.0, 13.0)	690/10.4 (9.4, 11.3)
	Unemployed	3,638/14.9 (14.3, 15.5)	973/16.0 (14.8, 17.2)	7,580/26.2 (25.5, 26.9)	1,809/27.3 (25.8, 28.7)
Household income					
	1st quintile (lowest)	1,665/5.8 (5.3, 6.3)	340/5.0 (4.2, 5.8)	2,593/7.8 (7.3, 8.3)	539/6.7 (5.7, 7.7)
	2nd quintile	1,964/8.3 (7.8, 8.8)	472/7.5 (6.6, 8.5)	2,670/9.2 (8.7, 9.7)	594/8.1 (7.0, 9.1)
	3rd quintile	2,229/10.8 (10.2, 11.4)	539/10.4 (9.5, 11.4)	2,771/10.6 (10.1, 11.1)	670/10.9 (9.9, 11.8)
	4th quintile	2,426/12.1 (11.6, 12.7)	604/12.2 (11.0, 13.5)	2,870/11.2 (10.7, 11.7)	739/12.4 (11.3, 13.4)
	5th quintile (highest)	2,597/12.8 (12.1, 13.6)	687/14.5 (12.8, 16.2)	2,967/11.4 (10.7, 12.1)	711/12.3 (10.8, 13.8)

Values are presented as number/weighted % (95% confidence interval). KNHANES, Korea National Health and Nutrition Examination Survey.

**Table 2.** Weighted prevalence of perceived severe stress in the KNHANES 2016-2019 sample (n=24,495) and in the KNHANES 2020 sample (n=5,854) in Korea

Variables		Total			Men			Women		
		2016-2019 (n=24,495)	2020 (n=5,854)	Difference (95% CI)	2016-2019 (n=10,767)	2020 (n=2,625)	Difference (95% CI)	2016-2019 (n=13,728)	2020 (n=3,229)	Difference (95% CI)
Adults ≥19 yr		4.9 (4.6, 5.2)	5.4 (4.6, 6.2)	0.5 (-0.4, 1.3)	4.1 (3.7, 4.5)	5.4 (4.2, 6.6)	1.3 (0.0, 2.5)^*^	5.7 (5.2, 6.2)	5.4 (4.6, 6.2)	-0.3 (-1.2, 0.6)
Age (yr)										
	19-29	7.5 (6.4, 8.6)	6.2 (4.5, 8.0)	-1.2 (-3.3, 0.9)	4.9 (3.6, 6.2)	5.4 (2.9, 7.8)	0.5 (-2.3, 3.3)	10.4 (8.6, 12.1)	7.1 (4.4, 9.9)	-3.2 (-6.4, 0.1)
	30-39	5.6 (4.8, 6.4)	9.2 (6.3, 12.1)	3.6 (0.6, 6.6)^*^	5.5 (4.3, 6.7)	9.5 (5.7, 13.3)	4.0 (0.0, 8.0)^*^	5.8 (4.6, 6.9)	8.9 (5.7, 12.1)	3.1 (-0.2, 6.5)
	40-49	4.1 (3.5, 4.8)	5.5 (3.9, 7.2)	1.4 (-0.4, 3.2)	4.0 (3.0, 5.0)	7.2 (4.5, 9.9)	3.2 (0.3, 6.1)^*^	4.3 (3.4, 5.2)	3.8 (1.9, 5.7)	-0.5 (-2.5, 1.6)
	50-59	4.1 (3.5. 4.8)	3.8 (2.5, 5.2)	-0.3 (-1.8, 1.1)	3.6 (2.8, 4.5)	3.4 (1.4, 5.4)	-0.2 (-2.4, 2.0)	4.6 (3.7, 5.5)	4.2 (2.6, 5.9)	-0.4 (-2.3, 1.5)
	60-69	4.2 (3.5, 4.9)	4.1 (2.5, 5.7)	-0.1 (-1.9, 1.6)	3.2 (2.3, 4.1)	3.3 (0.6, 6.1)	0.2 (-2.6, 3.1)	5.2 (4.2, 6.2)	4.8 (3.0, 6.6)	-0.4 (-2.5, 1.6)
	≥70	3.3 (2.7, 3.9)	2.9 (1.8, 4.0)	-0.4 (-1.7, 0.8)	2.0 (1.3, 2.8)	1.5 (0.4, 2.6)	-0.6 (-1.9, 0.8)	4.2 (3.4, 5.1)	3.9 (2.3, 5.6)	-0.3 (-2.2, 1.6)
Employment status										
	Employer	4.6 (3.9, 5.4)	4.5 (2.7, 6.3)	-0.1 (-2.1, 1.8)	4.0 (3.1, 4.9)	4.1 (1.9, 6.4)	0.1 (-2.2, 2.5)	6.2 (4.7, 7.7)	5.3 (2.4, 8.1)	-0.9 (-4.2, 2.3)
	Standard worker	4.8 (4.1, 5.5)	5.7 (4.0, 7.3)	0.9 (-0.9, 2.6)	3.8 (3.0, 4.5)	6.0 (3.9, 8.0)	2.2 (0.1, 4.4)^*^	7.0 (5.7, 8.4)	5.0 (3.0, 7.1)	-2.0 (-4.5, 0.5)
	Non-standard worker	4.9 (4.2, 5.6)	5.0 (3.6, 6.4)	0.2 (-1.4, 1.7)	4.3 (3.2, 5.4)	6.1 (3.4, 8.8)	1.9 (-1.0, 4.8)	5.4 (4.4, 6.4)	4.0 (2.6, 5.5)	-1.3 (-3.1, 0.4)
	Unemployed	5.1 (4.6, 5.6)	5.7 (4.6, 6.8)	0.6 (-0.6, 1.9)	4.4 (3.6, 5.3)	5.1 (3.0, 7.1)	0.6 (-1.6, 2.8)	5.4 (4.8, 6.1)	6.1 (4.9, 7.3)	0.7 (-0.7, 2.0)
Household income										
	1st quintile (lowest)	6.0 (5.1, 7.0)	6.4 (3.9, 8.9)	0.4 (-2.3, 3.0)	4.6 (3.4, 5.8)	7.9 (3.1, 12.8)	3.3 (-1.6, 8.3)	7.1 (5.8, 8.4)	5.2 (3.0, 7.5)	-1.9 (-4.5, 0.7)
	2nd quintile	4.6 (3.9, 5.3)	6.4 (4.4, 8.4)	1.8 (-0.3, 3.9)	3.8 (2.8, 4.9)	7.0 (3.8, 10.2)	3.1 (-0.1, 6.4)	5.2 (4.3, 6.2)	5.9 (3.4, 8.3)	0.6 (-2.0, 3.3)
	3rd quintile	4.6 (3.9, 5.3)	6.2 (4.5, 7.8)	1.5 (-0.3, 3.4)	3.5 (2.6, 4.4)	5.6 (3.1, 8.1)	2.1 (-0.5, 4.7)	5.8 (4.7, 6.8)	6.7 (4.5, 8.9)	0.9 (-1.5, 3.3)
	4th quintile	5.0 (4.3, 5.7)	5.3 (4.0, 6.6)	0.3 (-1.1, 1.8)	4.0 (3.1, 5.0)	5.2 (3.1, 7.2)	1.1 (-1.2, 3.4)	6.0 (4.9, 7.1)	5.5 (3.8, 7.2)	-0.5 (-2.5, 1.5)
	5th quintile (highest)	4.8 (4.1, 5.4)	3.9 (2.9, 4.9)	-0.9 (-2.1, 0.3)	4.6 (3.7, 5.5)	3.7 (2.1, 5.3)	-0.9 (-2.7, 0.9)	4.9 (4.0, 5.9)	4.1 (2.7, 5.5)	-0.9 (-2.6, 0.9)

Values are presented as weighted % (95% CI). KNHANES, Korea National Health and Nutrition Examination Survey; CI, confidence interval.

* p<0.05.

**Table 3.** Weighted prevalence of PHQ-9 detected depression in the KNHANES 2016, 2018 sample (n=11,679) and in the KNHANES 2020 sample (n=5,349) in Korea

Variables		Total			Men			Women		
		2016, 2018 (n=11,679)	2020 (n=5,349)	Difference (95% CI)	2016, 2018 (n=5,056)	2020 (n=2,417)	Difference (95% CI)	2016, 2018 (n=6,623)	2020 (n=2,932)	Difference (95% CI)
Adults ≥19 yr		4.9 (4.4, 5.4)	5.3 (4.5, 6.1)	0.4 (-0.5, 1.3)	3.2 (2.6, 3.8)	4.4 (3.4, 5.4)	1.2 (0.0, 2.3)^*^	6.6 (5.8, 7.4)	6.2 (5.2, 7.3)	-0.3 (-1.7, 1.0)
Age (yr)										
	19-29	6.0 (4.5, 7.5)	8.3 (6.2, 10.4)	2.4 (-0.2, 4.9)	3.2 (1.6, 4.8)	5.6 (3.0, 8.2)	2.4 (-0.6, 5.5)	9.1 (6. 4, 11.8)	11.3 (7.7, 15.0)	2.3 (-2.3, 6.8)
	30-39	4.8 (3.7, 6.0)	6.8 (4.6, 9.1)	2.0 (-0.4, 4.5)	3.6 (2.4, 4.9)	6.5 (3.8, 9.2)	3.0 (0.0, 6.0)^*^	6.1 (4.4, 7.9)	7.2 (4.0, 10.3)	1.0 (-2.6, 4.6)
	40-49	3.5 (2.6, 4.4)	4.9 (3.1, 6.6)	1.4 (-0.6, 3.3)	3.2 (2.0, 4.4)	5.5 (3.0, 8.0)	2.3 (-0.5, 5.1)	3.8 (2.6, 4.9)	4.2 (2.1, 6.3)	0.4 (-2.0, 2.8)
	50-59	4.1 (3.1, 5.0)	2.4 (1.4, 3.5)	-1.6 (-3.1, 0.2)	2.7 (1.5, 3.9)	1.9 (0.5, 3.3)	-0.8 (-2.6, 1.1)	5.4 (4.0, 6.8)	3 (1.4, 4.5)	-2.4 (-4.5, -0.3)
	60-69	5.4 (4.2, 6.5)	4.3 (2.9, 5.6)	-1.0 (-2.7, 0.7)	3.0 (1.9, 4.2)	3.1 (1.4, 4.7)	0.0 (-2.0, 2.1)	7.6 (5.7, 9.5)	5.4 (3.6, 7.2)	-2.0 (-4.5, 0.6)
	≥70	7.0 (5.7, 8.3)	5.3 (3.6, 7.1)	-1.7 (-3.9, 0.5)	3.8 (2.5, 5.1)	2.4 (0.8, 4.0)	-1.4 (-3.5, 0.6)	9.1 (7.2, 11.0)	7.5 (4.9, 10.2)	-1.6 (-4.9, 1.7)
Employment status										
	Employer	3.4 (2.5, 4.3)	3.2 (1.7, 4.7)	-0.2 (-1.9, 1.6)	2.2 (1.3, 3.1)	1.8 (0.5, 3.1)	-0.4 (-2.0, 1.2)	6.2 (4.1, 8.2)	6.1 (2.8, 9.5)	-0.1 (-4.0, 3.9)
	Standard worker	1.8 (1.2, 2.4)	3.4 (2.3, 4.6)	1.6 (0.3, 2.9)^*^	1.1 (0.6, 1.7)	3.5 (2.0, 5.1)	2.4 (0.8, 4.0)^*^	3.4 (2.0, 4.7)	3.2 (1.6, 4.8)	-0.2 (-2.3, 2.0)
	Non-standard worker	4.3 (3.3, 5.2)	3.9 (2.6, 5.3)	-0.3 (-2.0, 1.3)	3.1 (1.9, 4.3)	4.1 (2.2, 6.1)	1.1 (-1.2, 3.3)	5.2 (3.9, 6.5)	3.7 (1.7, 5.8)	-1.4 (-3.9, 1.0)
	Unemployed	7.9 (6.8, 8.9)	8.1 (6.6, 9.6)	0.3 (-1.6, 2.1)	6.8 (5.1, 8.6)	7.5 (4.9, 10.0)	0.7 (-2.4, 3.8)	8.4 (7.1, 9.6)	8.5 (6.6, 10.3)	0.1 (-2.1, 2.3)
Household income										
	1st quintile (lowest)	11.3 (9.4, 13.1)	10.7 (7.5, 13.9)	-0.5 (-4.1, 3.2)	9.8 (6.9, 12.6)	10.5 (5.1, 15.8)	0.9 (-5.2, 6.9)	12.4 (10.1, 14.7)	10.9 (7.3, 14.6)	-1.5 (-5.8, 2.8)
	2nd quintile	5.5 (4.4, 6.5)	5.1 (3.3, 7.0)	-0.3 (-2.4, 1.8)	3.2 (1.9, 4.5)	4.3 (1.9, 6.6)	1.1 (-1.6, 3.8)	7.5 (5.8, 9.2)	5.9 (3.5, 8.3)	-1.6 (-4.5, 1.4)
	3rd quintile	4.8 (3.7, 5.8)	6 (4.5, 7.5)	1.2 (-0.6, 3.0)	2.6 (1.5, 3.7)	4.3 (2.3, 6.3)	1.6 (-0.6, 3.9)	6.9 (5.2, 8.6)	7.6 (5.3, 10.0)	0.7 (-2.2, 3.6)
	4th quintile	3.1 (2.3, 3.8)	5.3 (4.0, 6.6)	2.2 (0.7, 3.7)^*^	1.6 (0.6, 2.5)	4.5 (2.4, 6.5)	2.9 (0.6, 5.1)^*^	4.7 (3.5, 5.9)	6.1 (4.2, 8.1)	1.4 (-0.8, 3.7)
	5th quintile (highest)	2.4 (1.6, 3.1)	2.8 (1.8, 3.9)	0.5 (-0.8, 1.8)	1.9 (1.0, 2.8)	2.5 (0.9, 4.1)	0.6 (-1.2, 2.4)	2.9 (1.7, 4.1)	3.2 (1.6, 4.8)	0.4 (-1.6, 2.3)

Values are presented as weighted % (95% CI). PHQ-9, Patient Health Questionnaire-9; KNHANES, Korea National Health and Nutrition Examination Survey; CI, confidence interval.

* p<0.05.

**Table 4.** Weighted prevalence of suicidal plans in the KNHANES 2016-2019 sample (n=24,502) and in the KNHANES 2020 sample (n=5,857) in Korea

Variables		Total			Men			Women		
		2016-2019 (n=24,502)	2020 (n=5,857)	Difference (95% CI)	2016-2019 (n=10,765)	2020 (n=2,626)	Difference (95% CI)	2016-2019 (n=13,737)	2020 (n=3,231)	Difference (95% CI)
Adults ≥19 yr		1.3 (1.1, 1.5)	1.8 (1.4, 2.1)	0.5 (0.1, 0.9)^*^	1.1 (0.9, 1.4)	1.5 (1.0, 2.1)	0.4 (-0.2, 1.0)	1.4 (1.2, 1.7)	2.0 (1.4, 2.5)	0.6 (-0.1, 1.2)
Age (yr)										
	19-29	1.2 (0.8, 1.6)	2.4 (1.3, 3.5)	1.2 (0.0, 2.3)^*^	0.9 (0.3, 1.5)	1.6 (0.5, 2.7)	0.7 (-0.6, 2.0)	1.6 (0.9, 2.2)	3.2 (1.2, 5.3)	1.7 (-0.5, 3.8)
	30-39	0.9 (0.5, 1.3)	1.9 (0.7, 3.1)	1.0 (-0.2, 2.3)	0.7 (0.3, 1.1)	2.1 (0.3, 4.0)	1.5 (-0.4, 3.3)	1.1 (0.5, 1.7)	1.7 (0.2, 3.1)	0.6 (-1.0, 2.1)
	40-49	0.7 (0.5, 1.0)	1.6 (0.7, 2.6)	0.9 (0.0, 1.8)^*^	0.8 (0.4, 1.2)	1.1 (0.1, 2.2)	0.4 (-0.8, 1.5)	0.7 (0.4, 1.1)	2.2 (0.6, 3.8)	1.5 (-0.1, 3.1)
	50-59	1.3 (0.9, 1.7)	1.3 (0.4, 2.2)	0.0 (-1.0, 0.9)	1.2 (0.6, 1.7)	1.5 (0.3, 2.6)	0.3 (-1.0, 1.6)	1.4 (0.9, 2.0)	1.1 (0.1, 2.1)	-0.3 (-1.5, 0.8)
	60-69	2.1 (1.6, 2.5)	1.7 (0.9, 2.4)	-0.4 (-1.3, 0.5)	2.1 (1.4, 2.8)	1.1 (0.3, 1.9)	-1.0 (-2.1, 0.1)	2.0 (1.4, 2.7)	2.1 (0.9, 3.4)	0.1 (-1.3, 1.5)
	≥70	2.1 (1.6, 2.6)	1.7 (1.0, 2.4)	-0.3 (-1.2, 0.6)	2.1 (1.4, 2.7)	1.9 (0.5, 3.4)	-0.1 (-1.7, 1.4)	2.1 (1.5, 2.7)	1.5 (0.7, 2.4)	-0.4 (-1.5, 0.7)
Employment status										
	Employer	0.8 (0.5, 1.1)	0.8 (0.1, 1.4)	0.0 (-0.7, 0.7)	0.7 (0.4, 1.1)	0.9 (-0.1, 1.8)	0.2 (-0.8, 1.1)	0.9 (0.3, 1.5)	0.6 (-0.1, 1.2)	-0.3 (-1.2, 0.6)
	Standard worker	0.4 (0.2, 0.5)	0.6 (0.2, 1.1)	0.2 (-0.2, 0.7)	0.4 (0.2, 0.6)	0.5 (0.0, 1.0)	0.1 (-0.4, 0.7)	0.3 (0.1, 0.6)	0.7 (-0.1, 1.6)	0.4 (-0.4, 1.3)
	Non-standard worker	1.2 (0.8, 1.5)	1.9 (1.0, 2.8)	0.8 (-0.2, 1.7)	1.3 (0.7, 1.8)	2.2 (0.9, 3.5)	0.9 (-0.5, 2.4)	1.1 (0.7, 1.4)	1.7 (0.3, 3.0)	0.6 (-0.8, 2.0)
	Unemployed	2.1 (1.8, 2.4)	2.6 (1.9, 3.4)	0.6 (-0.2, 1.4)	2.2 (1.6, 2.7)	2.5 (1.3, 3.7)	0.3 (-1.0, 1.6)	2.0 (1.6, 2.4)	2.7 (1.8, 3.6)	0.7 (-0.3, 1.7)
Household income										
	1st quintile (lowest)	3.4 (2.8, 4.1)	4.4 (2.8, 6.1)	1.0 (-0.8, 2.8)	3.5 (2.5, 4.5)	3.7 (1.2, 6.2)	0.2 (-2.5, 2.9)	3.4 (2.5, 4.2)	5.0 (2.8, 7.2)	1.6 (-0.7, 4.0)
	2nd quintile	1.6 (1.2, 2.0)	2.0 (1.0, 3.1)	0.5 (-0.7, 1.6)	1.5 (0.9, 2.1)	3.0 (1.1, 4.9)	1.4 (-0.6, 3.5)	1.6 (1.1, 2.1)	1.2 (0.3, 2.1)	-0.4 (-1.5, 0.6)
	3rd quintile	1.2 (0.9, 1.6)	1.7 (1.0, 2.5)	0.5 (-0.3, 1.4)	1.1 (0.6, 1.6)	1.4 (0.3, 2.4)	0.3 (-0.9, 1.4)	1.3 (0.8, 1.9)	2.1 (0.9, 3.3)	0.8 (-0.5, 2.0)
	4th quintile	0.7 (0.5, 1.0)	1.5 (0.8, 2.2)	0.8 (0.0, 1.5)^*^	0.7 (0.3, 1.1)	1.5 (0.5, 2.5)	0.8 (-0.3, 1.8)	0.8 (0.4, 1.2)	1.5 (0.3, 2.7)	0.7 (-0.5, 2.0)
	5th quintile (highest)	0.5 (0.3, 0.7)	0.7 (0.2, 1.3)	0.2 (-0.3, 0.8)	0.3 (0.1, 0.5)	0.3 (-0.1, 0.6)	0.0 (-0.4, 0.3)	0.7 (0.3, 1.0)	1.3 (0.1, 2.4)	0.6 (-0.6, 1.8)

Values are presented as weighted % (95% CI). KNHANES, Korea National Health and Nutrition Examination Survey; CI, confidence interval.

* p<0.05.

## SUPPLEMENTARY MATERIALS

Supplementary Material 1. Flowchart of the included analysis samples in each outcomes A: analysis for severe perceived stress; B: analysis for depression; C: analysis for suicidal plans
