# Supplementary material for: Mental health of Korean adults before and during the COVID-19 pandemic: a special report of the 2020 Korea National Health and Nutrition Examination Survey
Source: Epidemiol Health. 2022 Apr 25;44:e2022042. doi: 10.4178/epih.e2022042 (PMC9133599; doi:10.4178/epih.e2022042)
Supplement: Supplementary Material 1. — Flowchart of the included analysis samples in each outcomes A: analysis for severe perceived stress; B: analysis for depression; C: analysis for suicidal plans [file epih-44-e2022042-suppl.docx]

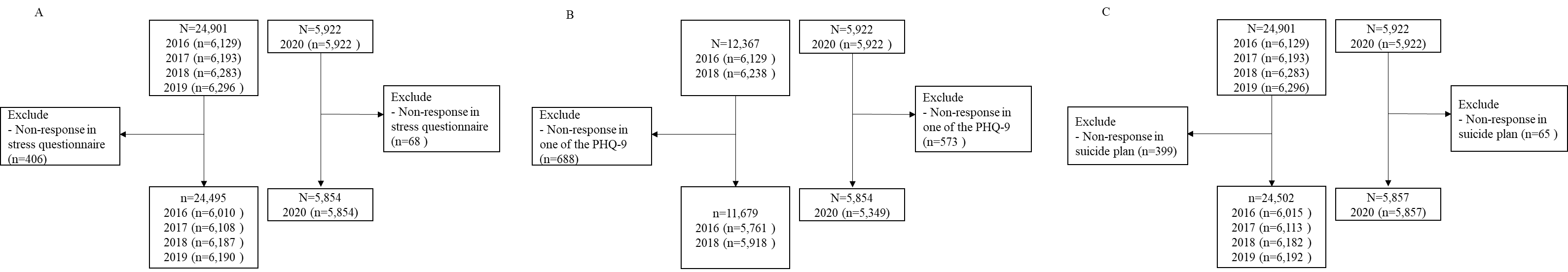


Supplementary Material 1. Flowchart of the included analysis samples in each outcomes

A: analysis for severe perceived stress; B: analysis for depression; C: analysis for suicidal plans
